# Clinical assessment of gait and functional mobility in Italian healthy and cognitively impaired older persons using wearable inertial sensors

**DOI:** 10.1007/s40520-020-01715-9

**Published:** 2020-09-25

**Authors:** Ilaria Mulas, Valeria Putzu, Gesuina Asoni, Daniela Viale, Irene Mameli, Massimiliano Pau

**Affiliations:** 1 Department of Mechanical, Chemical and Materials Engineering, University of Cagliari, Piazza d’Armi, 09123 Cagliari, Italy; 2 Center for Cognitive Disorders and Dementia, Geriatric Unit SS. Trinità Hospital, Via Romagna 16, 09127 Cagliari, Italy

**Keywords:** Gait, Timed-up-and-go (TUG), Functional mobility, Older adults, Accelerometer, Inertial measurement unit (IMU)

## Abstract

**Aim:**

The main purpose of the present study was to verify the feasibility of wearable inertial sensors (IMUs) in a clinical setting to screen gait and functional mobility in Italian older persons. In particular, we intended to verify the capability of IMUs to discriminate individuals with and without cognitive impairments and assess the existence of significant correlations between mobility parameters extracted by processing trunk accelerations and cognitive status.

**Methods:**

This is a cross-sectional study performed on 213 adults aged over 65 years (mean age 77.0 ± 5.4; 62% female) who underwent cognitive assessment (through Addenbrooke’s Cognitive Examination Revised, ACE-R) instrumental gait analysis and the Timed Up and Go (TUG) test carried out using a wearable IMU located in the lower back.

**Results:**

Individuals with cognitive impairments exhibit a peculiar gait pattern, characterized by significant reduction of speed (− 34% vs. healthy individuals), stride length (− 28%), cadence (− 9%), and increase in double support duration (+ 11%). Slight, but significant changes in stance and swing phase duration were also detected. Poorer performances in presence of cognitive impairment were observed in terms of functional mobility as overall and sub-phase TUG times resulted significantly higher with respect to healthy individuals (overall time, + 38%, sub-phases times ranging from + 22 to + 34%), although with some difference associated with age. The severity of mobility alterations was found moderately to strongly correlated with the ACE-R score (Spearman’s rho = 0.58 vs. gait speed, 0.54 vs. stride length, 0.66 vs. overall TUG time).

**Conclusion:**

The findings obtained in the present study suggest that wearable IMUs appear to be an effective solution for the clinical assessment of mobility parameters of older persons screened for cognitive impairments within a clinical setting. They may represent a useful tool for the clinician in verifying the effectiveness of interventions to alleviate the impact of mobility limitations on daily life in cognitively impaired individuals.

## Introduction

The progressive loss of ambulation and functional mobility performance of humans is a physiologic consequence of aging mainly due to reductions in muscle strength and deterioration of sensory, vestibular and proprioceptive inputs [1–3]. In presence of specific conditions such as obesity, musculoskeletal and neurologic disorders, depression, etc., reduction in mobility may result exacerbated. Similarly, cognitive disorders, whatever their nature, are known to impact gait, balance and mobility. In all these cases, the performance of even simple activities of daily living (ADL) is restricted [4], social participation reduced [5] and, in general, the overall quality of life is significantly compromised [6].

In the last decade, there has been a rising interest in investigating the role played by cognition on mobility, particularly as regards the relationship between cognitive functions and basic motor tasks such as gait and balance. For instance, it is now quite clear that although walking is mostly an automatic task, cognitive performances are strongly implicated in balance/postural control through management of axial musculature and integration of visual, vestibular, proprioceptive and sensory feedback. Moreover, this interplay is age-dependent, as different neural substrates are engaged in the execution of cognitive tasks in the young and the older adults [7]. When external conditions tend to reduce the automaticity of the task (i.e., uneven terrain, concurrent motor/cognitive tasks, existence of neurologic disorders), additional cognitive resources are needed, thus compromising gait performance and increasing instability [8].

Gait alterations in older persons, common even in absence of specific pathologies, are usually quantified in terms of changes occurring in variables associated with the gait cycle. For instance, modification of spatio-temporal parameters such as speed, stride length and cadence (which all tend to decrease with aging) have been often reported [9]. Even other aspects of walking performance such as symmetry, regularity, coordination, dynamic balance and foot movements, may provide information on specific (and sometimes subtle) gait dysfunctions [10, 11].

Recent studies suggest that gait parameters can be effectively employed as early clinical marker of cognitive decline and dementia, given that gait abnormalities may precede them by several years. In this regard, it has been observed that even in apparently healthy older persons, early disturbances in cognitive processes such as attention, executive functions and working memory often coexist with slower gait speed and greater instability [12, 13]. Verghese et al. [14] observed that in non-demented individuals, the simultaneous presence of subjective cognitive complaints and slow gait depict a predementia syndrome which they defined as Motoric Cognitive Risk (MCR) syndrome. Several subsequent studies have pointed out that older persons with MCR are at high-risk of dementia, exhibit more chronic illnesses and are subject to a range of adverse outcomes including disability and falls.

Poor cognitive performances influence not only gait, but also other motor tasks essential for the independence of the older person, such as rising from and sitting down in a chair, turning, etc. These can be even more demanding than gait in terms of cognitive resources required for planning, orientation in space and organization purposes [15]. The ability to perform such activities, which are commonly classified under the umbrella of “functional mobility”, can be easily assessed using a wide range of tools [16]. Among them, the Timed-Up-and-Go test (TUG, [17]) is one of the most common, owing to its clinical utility in diagnosing risk of falls [18, 19] in community-dwelling and frail older adults. However, TUG has been demonstrated also reliable in detecting functional mobility limitations among individuals with cognitive impairments and dementia at different stages [20].

In particular, it has been observed that older adults with cognitive impairments show a higher TUG time with respect to unaffected individuals [21, 22]. They also exhibit moderate to large correlations between cognitive performance (assessed using either Addenbrooke’s Cognitive Examination Revised ACE-R, [23, 24] or the Montreal Cognitive Assessment, MoCA, [25]) and overall TUG time or TUG sub-phase speed (in particular intermediate and final 180° turning time [23]). The results of a recent meta-analysis [20] suggest that TUG time might be effectively employed as a marker to support the diagnosis and identification of dementia stages, including the prodromal phase.

It is to be noted that the quantitative assessment of gait and functional mobility parameters in older persons with or without cognitive impairment are usually performed with a variety of methods ranging from the use of a simple stopwatch (suitable for calculating gait speed and recording TUG time) to more sophisticated equipment such as optical motion capture systems and electronic walkways that provide data on several spatio-temporal and kinematic parameters [26–28]. Nevertheless, while in principle a large set of quantitative, robust, and reliable data is desirable to accurately investigate mobility, it should also be considered that besides the cost and complexity of such systems, they often require dedicated space and specialized personnel. As a result, they are unsuitable for home- community- and ambulatory-based care [29].

In recent years, inertial measurement units (IMUs are devices composed of a tri-axial accelerometer, gyroscope and magnetometer) have become widespread in human movement analysis owing to their reliability, reduced cost and ease of use [25, 30]. Miniaturized wearable IMUs allow execution of a variety of tests on balance, gait and functional mobility under ecological conditions, and have already been employed to test older adults with and without cognitive impairments [31–36]. A simple setup consisting of a single unit placed on the lower back (widely used to test individuals with neurologic disorders, [37]) appears feasible for use in home and clinical settings since it requires a relatively short time to prepare the subject and perform the analysis. This approach also allows performance of a sort of instrumented version of clinical tests such as the 6- or 2-min walking test and TUG, while providing a larger amount of relevant information. For instance, an instrumented TUG provides data not only on the overall time required to perform it, but also time, speed and accelerations associated with each TUG sub-phase, namely sit-to-stand, intermediate and final 180° turns and stand-to-sit [34, 38–40]. Similarly, it is possible to extract several spatio-temporal parameters such as speed, cadence, step/stride length and duration of stance, swing and double support phases from a gait analysis assisted by IMUs. Moreover, further refined processing of trunk accelerations allows performance of more sophisticated analyses (i.e., those aimed at investigating stride-to-stride symmetry or “smoothness” of gait) [41–43], which may reveal slight changes in gait that occur even before they become detectable with conventional spatio-temporal parameters.

Based on the aforementioned considerations, the main purpose of the present study was to verify the feasibility of using IMUs in a clinical setting to screen gait and functional mobility in a cohort of community-dwelling older adults in a geriatric outpatient center specialized in diagnosis and treatment of cognitive disorders and dementia. In particular, we intended to verify the capability of IMU to discriminate, through the results of the instrumented gait and TUG test, individuals with or without cognitive impairments and assess the existence of significant correlations between cognitive status and mobility parameters. If confirmed, such findings would strengthen the idea of systematically employing quantitative analyses of mobility assisted by IMU in a clinical setting. Since IMU-based tests are relatively easy to perform, they might effectively integrate the conventional geriatric assessment and facilitate the early detection of signs of cognitive decline based on changes in gait and functional mobility.

## Methods

### Participants

In the period November 2019–February 2020, 213 adults aged over 65, consecutively examined at the Center for Cognitive Disorders and Dementia (in collaboration with the Geriatric Unit of “SS. Trinità” General Hospital, Cagliari, Italy) were enrolled in the study. Exclusion criteria were the presence of neurologic disorders able to interfere in mobility (e.g., Parkinson’s disease, multiple sclerosis and stroke), severe symptomatic orthopedic conditions and, in general, inability to walk independently. Individuals who needed aids to ambulate (i.e., canes, walking frames, crutches, etc.), were also excluded owing to the reduced reliability of instrumental measures of mobility for the specific setup employed herein [44].

Purposes and methodology of the study were carefully explained to all participants (or to their family members/caregivers when necessary) and they signed an informed consent form. They then underwent a detailed geriatric and psychological assessment during which their cognitive status was evaluated using the Italian version [45] of Addenbrooke’s Cognitive Examination Revised (ACE-R, [46]). ACE-R is articulated across five cognitive domains, namely attention and orientation, memory, verbal fluency (related to cognitive abilities of the executive function), visuospatial function, and language. The overall ACE-R score ranges from 0 to 100, with lower scores indicating superior cognitive impairment. The Italian version of ACE-R has been found reliable in discriminating individuals with or without mild dementia according to specific cut-offs calculated for young-old (< 75 years) and old-old (> 75 years) older persons [45]. These cut-offs were also employed in the present study to stratify participants into four groups as follows:

- Healthy controls young-old (age ≤ 75, HC-YO): ACE-R score ≥ 79 (*n* = 64)
- Healthy controls old-old (age > 75, HC-OO): ACE-R score ≥ 60 (*n* = 78)
- Cognitively impaired young-old (age ≤ 75, CI-YO): ACE-R score < 79 (*n* = 28)
- Cognitively impaired old-old (age > 75, CI-OO): ACE-R score < 60 (*n* = 43)

Their anthropometric and clinical features are reported in Table 1.

**Table 1:** Participants’ anthropometric and clinical features

	HC-YO	HC-OO	CI-YO	CI-OO
Participants # (F, M)	64 (43F, 21 M)	78 (44F, 34 M)	28 (19F, 9 M)	43 (27F, 16 M)
Age (years)	71.9 ± 2.3	80.7 ± 2.5	71.3 ± 2.9	81.5 ± 4.2
Body mass (kg)	66.1 ± 12.8	65.4 ± 12.1	62.5 ± 12.3	61.5 ± 14.6
Height (cm)	158.8 ± 7.5	160.5 ± 7.9	159.9 ± 9.5	157.0 ± 8.6
Years of education	11.6 ± 4.4	8.2 ± 4.5	8.3 ± 4.2	5.7 ± 2.7
Diagnoses and medical conditions, *n* (%)				
Depressive disorders	5 (8%)	9 (11%)	7 (25%)	8 (19%)
Diabetes and metabolic syndrome	18 (28%)	22 (29%)	13 (46%)	13 (30%)
Hypertension	25 (39%)	32 (41%)	13 (46%)	30 (70%)
Cardiovascular diseases	25 (38%)	27 (35%)	15 (53%)	27 (63%)
Musculoskeletal diseases	30 (46%)	36 (46%)	11 (40%)	25 (59%)
Routine prescription medications, *n* (%)				
Benzodiazepines	5 (8%)	22 (28%)	5 (18%)	5 (11%)
Beta-blockers	19 (23%)	22 (28%)	6 (21%)	11 (26%)
Antidepressants	5 (8%)	11 (14%)	8 (29%)	10 (22%)
Diuretics	7 (11%)	22 (28%)	8 (29%)	14 (33%)
Analgesics	0 (0%)	4 (5%)	2 (7%)	0 (0%)
Neuroleptics	5 (8%)	0 (0%)	8 (29%)	16 (37%)
ACE-R (overall)	90.9 ± 3.4	77.6 ± 7.3	57.6 ± 19.9	38.2 ± 17.1

Values are expressed as mean ± SD

*ACE-R* Addenbrooke’s Cognitive Examination (revised), *HC* healthy controls, *CI* cognitively impaired, *YO* young–old, *OO* old–old

The study was conducted in accordance with the ethical standards of the institutional research committee and the 1964 Helsinki declaration and its later amendments.

### Data acquisition and processing

Both gait and TUG tests were performed using a miniaturized wearable inertial sensor (G-Sensor^®^, BTS Bioengineering S.p.A., Italy) previously employed for similar investigations in older adults [40, 47–49], as well as in individuals with neurologic disorders [50, 51]. The sensor was attached to the individual’s trunk using a semi-elastic belt at two different positions which approximately corresponded to S1 vertebrae (for gait analysis) and L1 vertebrae (for TUG test) locations. Previous studies reported an overall good-to-excellent test–retest reliability for most parameters considered in the present study. This was true both for gait analysis [52, 53] and TUG [54, 55] performed with the same kind of setup, although some specific variables (in particular gait cycle phase duration and sit-to-stand time of TUG) should be interpreted with caution.

### Gait analysis

Participants were requested to walk along a 30-m hallway, following a straight trajectory at a self-selected speed and in the most natural manner. During the trial, the inertial sensor recorded accelerations along three orthogonal axes: antero-posterior (AP corresponding to the walking direction), medio-lateral (ML), and supero-inferior (V) at a frequency of 100 Hz. Data were transmitted in real-time via Bluetooth to a notebook, where they were later processed using a custom Matlab^®^ routine to calculate the following spatio-temporal parameters of gait: speed, stride length, cadence and duration of stance, swing and double support phase (expressed as a percentage of the gait cycle). In addition, the relationship between step length and cadence (i.e., walk ratio, [56, 57]) was calculated. It has been reported that the walk ratio is indicative of cautious gait, poor balance and impaired central control of gait and has also been associated with falls and cognitive performance in older persons [57, 58]. The gait parameters known to be influenced by an individual’s anthropometry (i.e., gait speed, stride length and cadence) were normalized by dividing them by each participant’s height [59–61]. Similarly, walk ratios were adjusted according to participant’s height following the approach proposed by Sekiya et al. [56]. Such procedures also allow removal of the effects of anthropometry on gait variables possibly associated with different M:F ratios of the four groups. In all acquisitions, the first and last two strides were excluded from the analysis to process data associated only with steady state conditions and thus remove the effects of acceleration and deceleration transients.

### Instrumented Timed Up and Go test

For the instrumented TUG (iTUG) tests, participants were requested to sit, with arms crossed at the wrists and held against the chest, on a standard office chair without armrests (seat height and width 48 cm, seat depth 40 cm) equipped with a back support 34 cm high. At the “start” signal, they stood up, walked for 3 m at a comfortable and safe speed [17], performed a 180° turn around a cone, walked back to the chair and performed a second 180° turn to sit down and end the test. In this case, two trials were performed: the first was to familiarize with the task and only the second was considered for the subsequent analysis. Since TUG is characterized by high test–retest reproducibility in older persons [62], a single trial can be considered sufficient to provide reliable data. Even in this case, accelerations were acquired at 100 Hz frequency and transmitted via Bluetooth to a notebook, where dedicated software (BTS G-Studio, BTS Bioengineering S.p.A., Italy) calculated the overall iTUG time and times associated with each sub-phase, namely:

- Sit-to stand: the transition from sitting to standing position
- Intermediate 180° turn: performed around the cone to invert the walking direction
- Final 180° turn: carried out to prepare the body to assume the sitting position at the end of the TUG
- Stand-to-sit: transition from sitting to standing position

### Statistical analysis

Differences in gait and iTUG parameters related to the cognitive status of participants were explored using one-way multivariate analysis of variance (MANOVA), where the independent variable was the group and the dependent variables the 7 gait parameters or the 5 iTUG parameters previously listed. In both cases, the level of significance was set at *p* = 0.05 and the effect size was assessed using the eta-squared (*η*^2^) coefficient. Univariate ANOVAs were carried out as a post hoc test by reducing the level of significance to *p* = 0.007 (0.05/7) for the gait analysis and to *p* = 0.01 (0.05/5) for the iTUG test after a Bonferroni correction for multiple comparisons. Where necessary, post hoc Holm–Sidak tests were performed to assess pairwise intra- and inter-group differences.

The relationship between gait/iTUG parameters and cognitive status (as indicated by the ACE-R score), was explored using Spearman’s rank correlation coefficient rho by setting the level of significance at *p* < 0.05. Rho values of 0.1, 0.3, and 0.5 were assumed to be representative of small, moderate, and large correlations respectively, according to Cohen’s guidelines [63]. All analyses were performed using the IBM SPSS Statistics v.20 software (IBM, Armonk, NY, USA).

## Results

The results of the experimental test for gait and the iTUG analysis are summarized in Tables 2 and 3. Table 4 reports the results of the correlation analysis between mobility features and ACE-R scores.

**Table 2:** Spatio-temporal parameters of gait measured with the inertial sensor. Values are expressed as mean ± SD

	HC-YO		HC-OO		CI-YO		CI-OO	
	Raw	Normalized	Raw	Normalized	Raw	Normalized	Raw	Normalized
Gait speed (m s^−1^)	1.10 ± 0.14	0.69 ± 0.09	0.95 ± 0.21^a^	0.59 ± 0.13 ^a^	0.79 ± 0.25^a,b^	0.50 ± 0.15 ^a^	0.60 ± 0.28^a,b,c^	0.38 ± 0.18^a,b,c^
Stride length (m)	1.16 ± 0.15	0.73 ± 0.09	1.05 ± 0.19^a^	0.65 ± 0.12	0.90 ± 0.25^a^	0.56 ± 0.15^a^	0.73 ± 0.29^a,b,c^	0.46 ± 0.18^a,b^
Cadence (steps min^−1^)	114.42 ± 8.28	72.25 ± 6.60	109.87 ± 9.65	68.69 ± 7.59	106.58 ± 13.31^a^	67.07 ± 10.43	98.88 ± 12.32^a,b^	63.16 ± 8.82^a,b^
Stance phase (% of the gait cycle)	60.19 ± 1.79		60.66 ± 2.07		61.11 ± 1.74		61.94 ± 2.12^a,b^	
Swing phase (% of the gait cycle)	39.81 ± 1.80		39.36 ± 2.04		38.87 ± 1.74		38.06 ± 2.15^a,b^	
Double support phase (% of the gait cycle)	20.42 ± 1.85		21.28 ± 2.00		22.24 ± 1.73		23.92 ± 2.10^a,b^	
Walk ratio [cm (steps min^−1^)^−1^]	0.51 ± 0.08		0.48 ± 0.09		0.42 ± 0.11^a^		0.37 ± 0.14^a,b^	

*HC* healthy controls, *CI* cognitively impaired, *YO* young–old, *OO* old–old, *iTUG* instrumented Timed Up and Go

^a^Significant difference vs. HC-YO after Bonferroni correction

^b^Significant difference vs. HC-OO after Bonferroni correction

^c^Significant difference vs. CI-YO after Bonferroni correction

**Table 3:** **i**TUG parameters measured with the inertial sensor

	HC-YO	HC-OO	CI-YO	CI-OO
iTUG duration (s)	11.1 ± 1.7	12.3 ± 2.0	15.0 ± 2.9^a^	21.7 ± 8.5^a,b,c^
Sit-to-stand time (s)	1.5 ± 0.3	1.5 ± 0.2	2.2 ± 1.1^a,b^	2.2 ± 0.9^a,b^
Intermediate 180° turn time (s)	2.2 ± 0.4	2.5 ± 0.7	2.7 ± 0.6	3.9 ± 1.4^a,b^
Final 180° turn time (s)	1.7 ± 0.5	2.0 ± 0.7	2.0 ± 0.6	2.7 ± 1.3^a,b,c^
Stand-to-sit time (s)	0.9 ± 0.3	0.9 ± 0.3	1.3 ± 0.6^a,b^	1.4 ± 0.6^a,b^

Values are expressed as mean ± SD

*HC* healthy controls, *CI* cognitively impaired, *YO* young–old, *OO* old–old, *iTUG* instrumented Timed Up and Go

^a^Significant difference vs. HC-YO after Bonferroni correction

^b^Significant difference vs. HC-OO after Bonferroni correction

^c^Significant difference vs. CI-YO after Bonferroni correction;

**Table 4:** Spearman’s coefficients for the correlations between ACE-R score and gait/functional mobility parameters

	Variables		Rho value
ACE-R vs	Gait parameters	Gait speed	0.580^†^
		Stride length	0.538^†^
		Cadence	0.436^†^
		Stance phase	− 0.294^†^
		Swing phase	0.293^†^
		Double support phase	− 0.286^†^
		Walk ratio	0.406^†^
	iTUG parameters	iTUG duration	− 0.662^†^
		Sit-to-stand time	− 0.382^†^
		Intermediate 180° turn time	− 0.473^†^
		Final 180° turn time	− 0.329^†^
		Stand-to-sit time	− 0.350^†^

*ACE-R* Addenbrooke’s Cognitive Examination (revised), *iTUG* instrumented Timed Up and Go

^†^*p* < 0.001

### Gait analysis

**Fig. 1:**
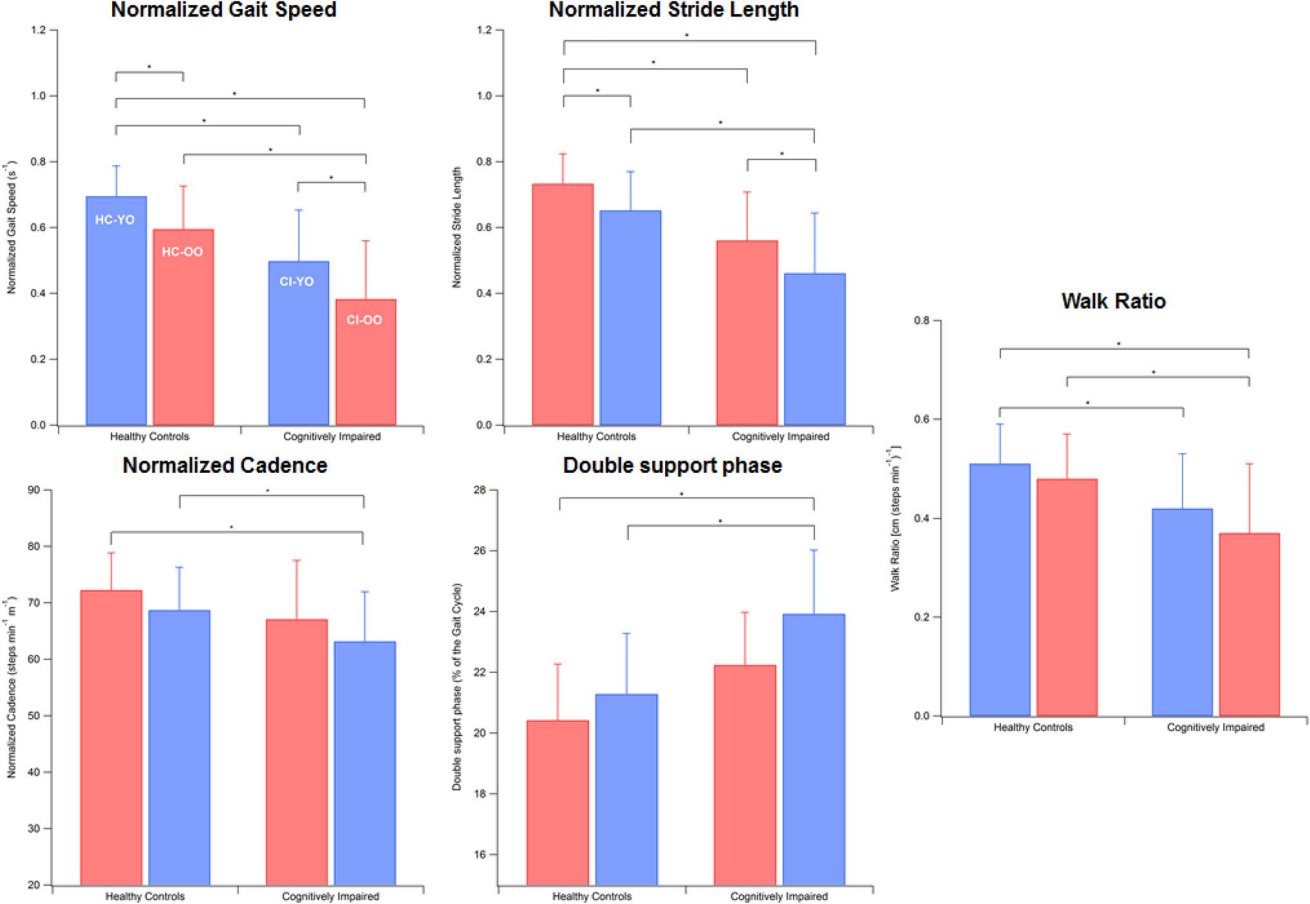
Trend of gait parameters across the groups tested. Error bars indicate standard deviation

MANOVA detected a significant main effect of group on gait parameters [*F*(18, 577.48) = 7.37, *p* < 0.001, Wilks *λ* = 0.56, *η*^2^ = 0.18]. As reported in Table 2 (and graphically shown in Fig. 1), all parameters exhibit a monotonic decrease on passing from the group of youngest individuals cognitively intact to the oldest participants cognitively impaired.

In particular, the post hoc analysis revealed that the CI-OO group exhibited gait speed values significantly lower than any other group (*p* = 0.003 vs. CI-YO, *p* < 0.001 vs. HC-OO and HC-YO) while stride length and cadence were reduced with respect to the HC-OO and CI-YO groups (*p* < 0.001). Similarly, stance and double support phases had increased in CI-OO with respect to HC-OO and HC-YO (*p* = 0.005 and *p* < 0.001 respectively) and, correspondingly, swing phase was reduced. The CI-YO group was characterized by reduced gait speed and stride length with respect to HC-YO (*p* < 0.001) and healthy individuals of different age ranges differed only as regards gait speed (*p* < 0.001). Walk ratio values of CI-OO were found significantly reduced with respect to HC-OO (*p* < 0.001) and HC-YO (*p* < 0.001), while those of CI-YO were lower with respect to HC-YO only (*p* = 0.001).

### iTUG test

**Fig. 2:**
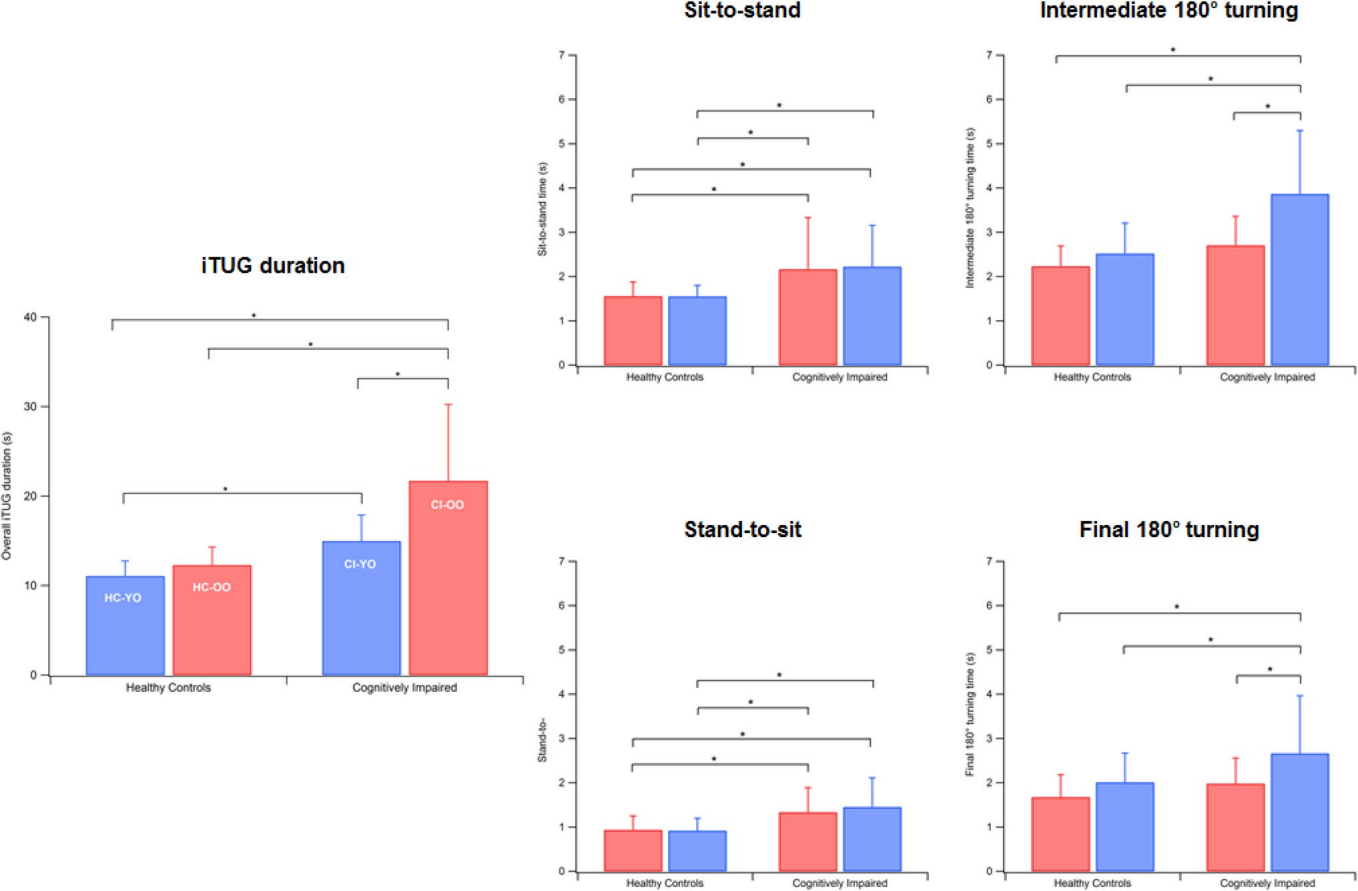
Trend of iTUG parameters across the groups tested. Error bars indicate standard deviation

Even for functional mobility (see data in Table 3), the statistical analysis detected a significant main effect of group on iTUG parameters [F(15, 566.32) = 12.93, *p* < 0.001, Wilks *λ* = 0.44, *η*^2^ = 0.24]. As shown in the diagrams in Fig. 2, a clear increasing trend, passing from the young-old healthy participants to the old-old cognitively impaired, is visible as regards overall iTUG time and 180° turns, while in the cases of sit-to-stand and stand-to-sit, differences are much less marked. In both healthy and cognitively impaired groups, the time necessary to stand and sit was very similar across the age groups, even though cognitively impaired people are generally slower in performing both tasks.

The post hoc analysis showed that cognitively impaired old-old participants exhibited poorer iTUG duration and final 180° turning times with respect to all other groups (*p* < 0.001 in all cases). In particular, the overall iTUG duration in the CI-OO group was almost double with respect to their cognitively intact age-matched peers (21.7 s vs. 12.3, *p* < 0.001). CI-OOs exhibited significantly higher sit-to-stand, intermediate 180° turning and stand-to-sit times with respect to HC-OOs and CI-YOs (*p* < 0.001). Cognitively impaired young-old participants exhibited significantly higher sit-to-stand and stand-to-sit times with respect to both groups of healthy controls and overall iTUG duration was higher than for HC-OOs. In conclusion, no differences were found in any parameter among healthy controls regardless of their age range.

### Correlation between mobility and cognitive status

Generally speaking, all mobility measures we investigated were found significantly correlated with cognitive status as expressed by the ACE-R score, although with some differences in magnitude. In particular, large positive correlations were observed between ACE-R and gait speed (rho = 0.580, *p* < 0.001) and stride length (rho = 0.536, *p* < 0.001), while a large negative correlation was observed with overall iTUG duration (rho = − 0.662, *p* < 0.001). Duration of gait cycle phases were found moderately correlated with ACE-R (rho values were slightly below 0.3, negative for stance and double support phase and positive for swing phase duration) while in the case of cadence and walk ratio, rho was 0.436 and 0.407, respectively (*p* < 0.001 in both cases). As regards iTUG, all parameters associated with the sub-phases were found negatively correlated with cognitive status, with rho values ranging from − 0.473 (intermediate 180° turn time) and − 0.329 (final 180° intermediate turning time).

### Practical considerations on measurements and possible pitfalls

IMUs represent an interesting solution in assessing mobility in older persons with and without cognitive impairment in a clinical setting for several reasons. First of all, differently from what occurs with laboratory-based motion capture systems, dedicated space/personnel are not required, the preparation of individuals is simple (i.e., no undressing or marker positioning phases) and thus they can perform the test immediately. Moreover, the positioning of the sensor is simple and fast, as the wireless connection with a notebook. The only critical point is represented by the autocalibration of the device, which is performed before the start of the trial: it lasts 4–5 s and requires participants to stay as still as possible. However, in our experience, the whole testing process (from device placement to data download and verification), including a brief familiarization phase, requires no more than 15–20 min for both gait and TUG tests. Data processing is immediate in the case of TUG, while 5 more minutes are necessary to export IMU data into a text file and then process it with the Matlab routine.

However, it must be recalled that the validity and reliability of IMU-based gait data may be affected by several factors, including random inclination changes of the sensor during walking, which may influence the results if not properly corrected [64]. Similarly, segmentation of the TUG phases may represent a critical issue, particularly as regards the definition of onset and offset of turns [38]. Finally, reliability of results can be greatly reduced when people who use walking aids are tested [44] and this would exclude from assessment a not-negligible part of the population.

## Discussion

The aim of this study was to assess the feasibility of using wearable IMUs in a “real-world” clinical setting to assess gait and functional mobility in older adults who underwent a geriatric screening for cognitive disorders. Another aim was to explore the relationship between mobility parameters instrumentally determined and cognitive status as expressed by the ACE-R score. Overall, our data suggest that IMUs can effectively describe changes in mobility associated with the presence of cognitive impairments.

The results of the gait analysis appear to reveal a clear pattern of ambulation for individuals with cognitive deficits, regardless of age, which is characterized by reduced speed, stride length and cadence. Such findings are in agreement with previous studies that demonstrated the existence of a specific motor signature associated with the presence of cognitive impairments [65], which has been hypothesized as attributable to a shared pathogenesis in executive functions, memory and gait decline. In older participants, we also observed additional alterations involving the subdivision of gait cycle phases, namely reduced swing phase and increased stance and double support phase duration, as well as significantly reduced walk ratio.

Reduction in gait speed represents the most distinctive feature associated with cognitive decline. For this reason, but also because it is easy to measure, speed is analyzed in most studies on gait of older persons at risk of Mild Cognitive Impairment (MCI) and dementia [8, 66, 67]. In this context, our data are fully consistent with existing literature, which almost unanimously indicates a strong relationship between gait speed and cognitive status (see the recent review and meta-analysis by Peel et al. for details [68]). Moreover, the average speed reduction observed herein (0.31 m/s for young-old and 0.35 m/s for old-old participants) can be considered clinically meaningful [68].

However, it must be recalled that aging itself, besides the presence of a coexisting cognitive disorder, causes speed reduction and thus the two effects are probably in some way superposed. In our sample, the influence of aging on speed was estimated at a 16% reduction for healthy participants, a value consistent with previous studies, which reported a speed decline in the range of 7–18% for the same age group [69–71]. This figure rose to 30% in our participants with cognitive impairment, thus indicating that, on average, approximately one half of the speed change could be attributed to cognitive deficit. It is also to be noted that the observed changes in gait speed due to the presence of cognitive deficits are age-dependent, as old-old individuals experience a more severe reduction with respect to young-old ones (− 58 vs. − 39%). The presence of significant alterations in other spatio-temporal parameters such as stride length (reduced in people with cognitive impairment) and duration of stance/double support phase, also suggest that individuals attempt to adapt their gait pattern to alterations in sensory or motor systems to achieve more stable locomotion and reduce the risk of falls [72]. Similarly, the walk ratio values calculated for both young and old cognitively impaired (significantly lower than their unaffected peers) are in agreement, even from a quantitative point of view, with those previously reported for individuals with mild to moderate dementia [73]. In such a context, low walk ratios are indicative of a strategy to compensate for the loss of gait stability [57].

The results of the iTUG test show that cognitive impairment is associated with higher overall TUG time, sit-to-stand and stand-to-sit times, regardless of the individual’s age, while only in older participants did we observe increased turning times. These findings suggest that coordination abilities, which are essential to performing optimal turns, are not greatly influenced by the presence of cognitive impairments when the individual is relatively young, while they always significantly affect TUG phases, which rely more on postural control and lower limb strength. This is likely due to a reduction in muscle strength (which was previously observed in individuals with MCI of similar age [74, 75]), and is also probably influenced by a limited amount of physical activity [76] and poor balance abilities [77]. It was also observed that old-old participants with cognitive impairments required longer times to perform 180° turns, as they probably adopted a cautious strategy to avoid loss of balance and falls. Such findings, observed in previous studies, are the consequence of a deficit in lower limb coordination [78] and poor performance in visual-spatial function and memory [79].

The results of the correlation analysis suggest that the level of cognitive impairment, as assessed by the ACE-R score, plays a relevant role in mobility performance, consistent with what is reported in literature. For instance, the review and meta-analysis by Demnitz et al. [80], which summarizes the results of 26 studies involving 26,000 participants, pointed out that speed (for gait) and TUG time (for functional mobility) are the variables more strongly associated with cognition measures such as the Mini Mental State Examination (MMSE), the Trail Making Test (TMT) Stroop, and the Verbal fluency and Digit Span.

The strong correlations found between ACE-R score and gait speed (0.580) and overall iTUG time (− 0.662) are partly consistent with previous studies [17, 18] which reported coefficient values in the range 0.36–0.60 for speed (depending on the degree of cognitive impairment of the tested subjects) and − 0.21 to − 0.68 for TUG time. Unfortunately, no correlation data are available for the remaining gait and iTUG parameters with ACE-R, but it is noteworthy that the recent study by Choi et al. [33] detected significant correlations between cadence, double support phase duration and stride length with cognitive status (assessed using MoCA), which is similar to our findings. Moreover, a recent study by Lee et al. [58] reported the existence of a significant moderate correlation between walk ratio and MMSE score in individuals with dementia. Such findings are in agreement with those presented here, which indicate a similar trend for correlation between walk ratio and ACE-R score.

Some limitations of the study are to be acknowledged. Firstly, we did not consider the effect of overweight/obesity (which were present in 42 and 11% of participants respectively), although such conditions are known to have a certain impact on mobility [81, 82]. Secondly, we had no information about other variables known to influence motor control in gait and functional mobility, such as actual levels of physical activity, fall-related psychological concerns or number of falls that occurred prior to the tests. Finally, in the analysis we did not include factors such as education, occupational status and type, wealth, etc., which might, to some extent, affect several aspects of mobility, especially in individuals younger than 70 [70, 83, 84] and thus the generalization of our results to different socio-economic contexts should be performed cautiously.

## Conclusion

Based on the findings of the present study, wearable IMUs appear to be a very effective solution for the assessment of mobility parameters of older persons screened for cognitive impairments within a clinical setting. As detailed information on a large set of gait and TUG parameters is available, it is possible to accurately define which aspects of mobility are more impaired in presence of a cognitive deficit. Data provided by such devices are useful not only to integrate the geriatric and neuropsychological assessment (and thus have a broader and more detailed view of the status of the older person) but can also help clinicians to plan specific psychoeducational interventions for caregivers and families and define tailored rehabilitation programs. Moreover, IMU-based data may support a better evaluation of the effectiveness of interventions aimed to alleviate the impact on daily life of mobility limitations in cognitively impaired individuals.

The results obtained in the present study indicate a well-defined framework of mobility alterations in cognitively impaired individuals, especially in the old-old group, expressed in the form of peculiar gait patterns characterized by reduced speed, stride length, cadence and swing phase duration, increased stance and double support duration, and altered coordination. The latter has a strong impact on simple motor tasks such as sitting/standing transition and turns. Some of these signs were also observed in young-old participants, even though the whole mobility pattern appeared slightly less compromised.

The severity of mobility alterations was found moderately to strongly correlated with the extent of the cognitive impairment, especially for gait speed, stride length and TUG duration, which were previously recognized as those mostly co-existing with mild cognitive impairments and dementia. However, the deeper analysis made possible by the IMUs showed that cognitive performance is also associated with gait cycle phase duration, thus indicating increased instability and fear of falling and with motor tasks, such as turns, which require a good level of coordination.

## References

[1] Rantakokko M, Mänty M, Rantanen T (2013). Mobility decline in old age. Exerc Sport Sci Rev.

[2] Iosa M, Fusco A, Morone G (2014). Development and decline of upright gait stability. Front Aging Neurosci.

[3] Ebeling PR, Cicuttini F, Scott D (2019). Promoting mobility and healthy aging in men: a narrative review. Osteoporos Int.

[4] Vermeulen J, Neyens JC, van Rossum E (2011). Predicting ADL disability in community-dwelling elderly people using physical frailty indicators: a systematic review. BMC Geriatr.

[5] Rosso AL, Taylor JA, Tabb LP (2013). Mobility, disability, and social engagement in older adults. J Aging Health.

[6] Davis JC, Bryan S, Best JR (2015). Mobility predicts change in older adults’ health-related quality of life: evidence from a Vancouver falls prevention prospective cohort study. Health Qual Life Out.

[7] Li KZH, Bherer L, Mirelman A (2018). Cognitive involvement in balance, gait and dual-tasking in aging: a focused review from a neuroscience of aging perspective. Front Neurol.

[8] Cohen JA, Verghese J, Zwerling JL (2016). Cognition and gait in older people. Maturitas.

[9] Prince F, Corriveau H, Hébert R (1997). Gait in the elderly. Gait Posture.

[10] Lindemann U (2020). Spatiotemporal gait analysis of older persons in clinical practice and research: which parameters are relevant?. Z Gerontol Geriatr.

[11] Hollman JH, McDade EM, Petersen RC (2011). Normative spatiotemporal gait parameters in older adults. Gait Posture.

[12] Montero-Odasso M, Verghese J, Beauchet O (2012). Gait and cognition: a complementary approach to understanding brain function and the risk of falling. J Am Geriatr Soc.

[13] Amboni M, Barone P, Hausdorff JM (2013). Cognitive contributions to gait and falls: evidence and implications: cognitive contributions to gait and falls. Mov Disord.

[14] Verghese J, Wang C, Lipton RB (2013). Motoric cognitive risk syndrome and the risk of dementia. J Gerontol A Biol Sci Med Sci.

[15] Herman T, Giladi N, Hausdorff JM (2011). Properties of the 'timed up and go' test: more than meets the eye. Gerontology.

[16] Soubra R, Chkeir A, Novella JL (2019). A systematic review of thirty-one assessment tests to evaluate mobility in older adults. Biomed Res Int.

[17] Podsiadlo D, Richardson S (1991). The Timed “Up & Go”: a test of basic functional mobility for frail elderly persons. J Am Geriatr Soc.

[18] Shumway-Cook A, Brauer S, Woollacott M (2000). Predicting the probability for falls in community-dwelling older adults using the Timed Up & Go test. Phys Ther.

[19] Christopher A, Kraft E, Olenick H (2019). The reliability and validity of the timed Up and Go as a clinical tool in individuals with and without disabilities across a lifespan: a systematic review: psychometric properties of the Timed Up and Go. Disabil Rehabil.

[20] de Oliveira SF, Ferreira JV, Plácido J (2019). Stages of mild cognitive impairment and Alzheimer’s disease can be differentiated by declines in Timed Up and Go test: a systematic review and meta-analysis. Arch Gerontol Geriatr.

[21] Ibrahim A, Singh DKA, Shahar S (2017). ‘Timed Up and Go’ test: age, gender and cognitive impairment stratified normative values of older adults. PLoS ONE.

[22] Rajtar-Zembaty A, Rajtar-Zembaty J, Sałakowski A (2019). Global cognitive functioning and physical mobility in older adults with and without mild cognitive impairment: evidence and implications. Folia Med Cracov.

[23] Ansai JH, Andrade LP, de Nakagawa TH (2017). Cognitive correlates of timed up and go subtasks in older people with preserved cognition mild cognitive impairment, and Alzheimer’s disease. Am J Phys Med Rehabil.

[24] de Melo LM, Ansai JH, Giusti Rossi P (2019). Performance of an adapted version of the Timed Up-and-Go test in people with cognitive impairments. J Mot Behav.

[25] Van Patten R, Lee E, Graham S et al (2019) The utility of the Timed Up-and-Go test in predicting cognitive performance: a cross-sectional study of independent living adults in a retirement community. J App Gerontol 39(10):1163–1168. 10.1177/0733464819872636 10.1177/0733464819872636 PMC7490461 32924758

[26] Snijders AH, van de Warrenburg BP, Giladi N (2007). Neurological gait disorders in elderly people: clinical approach and classification. Lancet Neurol.

[27] Bridenbaugh SA, Kressig RW (2011). Laboratory review: the role of gait analysis in seniors’ mobility and fall prevention. Gerontol.

[28] Wren TAL, Gorton GE, Õunpuu S (2011). Efficacy of clinical gait analysis: a systematic review. Gait Posture.

[29] Zhong R, Rau PLP (2020). Are cost-effective technologies feasible to measure gait in older adults? A systematic review of evidence-based literature. Arch Gerontol Geriatr.

[30] Iosa M, Picerno P, Paolucci S (2016). Wearable inertial sensors for human movement analysis. Expert Rev Med Devices.

[31] Culhane KM, O’Connor M, Lyons D (2005). Accelerometers in rehabilitation medicine for older adults. Age Ageing.

[32] Maquet D, Lekeu F, Warzee E (2010). Gait analysis in elderly adult patients with mild cognitive impairment and patients with mild Alzheimer’s disease: simple versus dual task: a preliminary report. Clin Physiol Funct Imaging.

[33] Choi JS, Oh HS, Kang DW (2011). Comparison of gait and cognitive function among the elderly with Alzheimer’s disease, mild cognitive impairment and healthy. Int J Precis Eng Manuf.

[34] Mirelman A, Weiss A, Buchman AS (2014). Association between performance on Timed Up and Go subtasks and mild cognitive impairment: further insights into the links between cognitive and motor function. J Am Geriatr Soc.

[35] Grimm B, Bolink S (2016). Evaluating physical function and activity in the elderly patient using wearable motion sensors. EFORT Open Rev.

[36] Mc Ardle R, Del Din S, Galna B (2020). Differentiating dementia disease subtypes with gait analysis: feasibility of wearable sensors?. Gait Posture.

[37] Brognara L, Palumbo P, Grimm B (2019). Assessing gait in Parkinson’s disease using wearable motion sensors: a systematic review. Diseases.

[38] Salarian A, Horak FB, Zampieri C (2010). ITUG, a sensitive and reliable measure of mobility. IEEE Trans Neural Syst Rehabil Eng.

[39] Zakaria NA, Kuwae Y, Tamura T (2015). Quantitative analysis of fall risk using TUG test. Comput Methods Biomech Biomed Eng.

[40] Porta M, Pilloni G, Corona F (2018). Relationships between objectively assessed functional mobility and handgrip strength in healthy older adults. Eur Geriatr Med.

[41] Brach JS, McGurl D, Wert D (2011). Validation of a measure of smoothness of walking. J Gerontol A Biol Sci Med Sci.

[42] Lowry KA, Lokenvitz N, Smiley-Oyen AL (2012). Age- and speed-related differences in harmonic ratios during walking. Gait Posture.

[43] Kikkert LHJ, Vuillerme N, van Campen JP (2017). Gait characteristics and their discriminative power in geriatric patients with and without cognitive impairment. J NeuroEng Rehabil.

[44] Zijlstra W (2004). Assessment of spatio-temporal parameters during unconstrained walking. Eur J Appl Physiol.

[45] Pigliautile M, Ricci M, Mioshi E (2011). Validation study of the Italian Addenbrooke’s cognitive examination revised in a young-old and old-old population. Dement Geriatr Cogn Disord.

[46] Mioshi E, Dawson K, Mitchell J (2006). The Addenbrooke’s Cognitive Examination Revised (ACE-R): a brief cognitive test battery for dementia screening. Int J Geriatr Psychiatry.

[47] Pau M, Porta M, Pilloni (2018). Texting while walking induces gait pattern alterations in healthy older adults. Hum Factors Ergon Soc Annu.

[48] Fastame MC, Hitchcott PK, Corona F (2019). Memory, subjective memory and motor functioning in non-demented elders with and without Parkinson’s disease. Eur J Psychol.

[49] Mangano GRA, Valle MS, Casabona A (2020). Age-related changes in mobility evaluated by the Timed Up and Go test instrumented through a single sensor. Sensors.

[50] Galli M, Kleiner A, Gaglione M (2015). Timed Up and Go test and wearable inertial sensor: a new combining tool to assess change in subject with Parkinson’s disease after automated mechanical peripheral stimulation treatment. Int J Eng Innov Technol.

[51] Pau M, Caggiari S, Mura A (2016). Clinical assessment of gait in individuals with multiple sclerosis using wearable inertial sensors: comparison with patient-based measure. Mult Scler Relat Dis.

[52] De Ridder R, Lebleu J, Willems T (2019). Concurrent validity of a commercial wireless trunk triaxial accelerometer system for gait analysis. J Sport Rehabil.

[53] Vítecková S, Horáková H, Poláková K (2020). Agreement between the GAITRite system and the wearable sensor BTS G-Walk for measurement of gait parameters in healthy adults and Parkinson’s disease patients. PeerJ.

[54] van Lummel RC, Walgaard S, Hobert MA (2016). Intra-rater, inter-rater and test–retest reliability of an instrumented Timed Up and Go (iTUG) test in patients with Parkinson's disease. PLoS ONE.

[55] Kleiner AFR, Pacifici I, Vagnini A (2018). Timed Up and Go evaluation with wearable devices: validation in Parkinson's disease. J Bodyw Mov Ther.

[56] Sekiya NNH, Ito H, Furuna T (1996). The invariant relationship between step length and step rate during free walking. J Hum Mov Stud.

[57] Bogen B, Moe-Nilssen R, Ranhoff AH (2018). The walk ratio: investigation of invariance across walking conditions and gender in community-dwelling older people. Gait Posture.

[58] Lee NG, Kang TW, Park HJ (2020). Relationship between balance, gait, and activities of daily living in older adults with dementia. Geriatr Orthop Surg Rehabil.

[59] Beck RJ, Andriacchi TP, Kuo KN (1981). Changes in the gait patterns of growing children. JBJS.

[60] Stansfield B, Hawkins K, Adams S (2018). A mixed linear modelling characterisation of gender and speed related changes in spatiotemporal and kinematic characteristics of gait across a wide speed range in healthy adults. Med Eng Phys.

[61] Dini P, David A (2009). Repetibilidade dos parametros espaço-temporais da marcha: Comparaçao entre crianças normais e com paralisia cerebral do tipo hemiplegia espastica. Braz J Phys Ther.

[62] Rydwik E, Bergland A, Forsén L (2011). Psychometric properties of Timed Up and Go in elderly people: a systematic review. Phys Occup Ther Geriatr.

[63] Cohen J (1992). Statistical power analysis. Curr Dir Psychol Sci.

[64] Trojaniello D, Cereatti A, Della CU (2014). Accuracy, sensitivity and robustness of five different methods for the estimation of gait temporal parameters using a single inertial sensor mounted on the lower trunk. Gait Posture.

[65] Montero-Odasso M, Oteng-Amoako A, Speechley M (2014). The motor signature of mild cognitive impairment: results from the gait and brain study. J Gerontol A Biol Sci Med Sci.

[66] Buracchio T, Dodge HH, Howieson D (2010). The trajectory of gait speed preceding mild cognitive impairment. Arch Neurol.

[67] Grande G, Triolo F, Nuara A (2019). Measuring gait speed to better identify prodromal dementia. Exp Gerontol.

[68] Peel NM, Alapatt LJ, Jones LV (2019). The association between gait speed and cognitive status in community-dwelling older people: a systematic review and meta-analysis. J Gerontol A Biol Sci Med Sci.

[69] Ferrucci L, Cooper R, Shardell M (2016). Age-related change in mobility: perspectives from life course epidemiology and geroscience. J Gerontol A Biol Sci Med Sci.

[70] Weber D (2016). Differences in physical aging measured by walking speed: evidence from the English Longitudinal Study of Ageing. BMC Geriatr.

[71] Stringhini S, Carmeli C, Jokela M (2018). Socioeconomic status, non-communicable disease risk factors, and walking speed in older adults: multi-cohort population-based study. BMJ.

[72] Salzman B (2010). Gait and balance disorders in older adults. Am Fam Physician.

[73] Schwenk M, Zieschang T, Englert S (2014). Improvements in gait characteristics after intensive resistance and functional training in people with dementia: a randomised controlled trial. BMC Geriatr.

[74] Auyeung TW, Kwok T, Lee J (2008). Functional decline in cognitive impairment; the relationship between physical and cognitive function. Neuroepidemiology.

[75] Caldas ÉC, Rezende LA, Oliveira KS (2017). Muscle strength, lower extremity functional performance and body composition in elderly women with mild cognitive impairment. Fisioter Mov.

[76] Geda YE, Roberts RO, Knopman DS (2010). Physical exercise, aging, and mild cognitive impairment: a population-based study. Arch Neurol.

[77] Franssen EH, Somen LEM, Torossian CL (1999). Equilibrium and limb coordination in mild cognitive impairment and mild Alzheimer’s disease. J Am Geriatr Soc.

[78] Boyle PA, Wilson RS, Buchman AS (2007). Lower extremity motor function and disability in mild cognitive impairment. Exp Aging Res.

[79] Mancini M, Schlueter H, El-Gohary M (2016). Continuous monitoring of turning mobility and its association to falls and cognitive function: a pilot study. J Gerontol A Biol Sci Med Sci.

[80] Demnitz N, Esser P, Dawes H (2016). A systematic review and meta-analysis of cross-sectional studies examining the relationship between mobility and cognition in healthy older adults. Gait Posture.

[81] Hergenroeder AL, Wert DM, Hile ES (2011). Association of body mass index with self-report and performance-based measures of balance and mobility. Phys Ther.

[82] Runhaar J, Koes BW, Clockaerts S (2011). A systematic review on changed biomechanics of lower extremities in obese individuals: a possible role in development of osteoarthritis: obese biomechanics of everyday movements. Obes Rev.

[83] Zaninotto P, Sacker A, Head J (2013). Relationship between wealth and age trajectories of walking speed among older adults: evidence from the English Longitudinal Study of Ageing. J Gerontol A Biol Sci Med Sci.

[84] Busch TA, Duarte YA, Pires Nunes D (2015). Factors associated with lower gait speed among the elderly living in a developing country: a cross-sectional population-based study. BMC Geriatr.

